# Fate and Removal of Antibiotics and Antibiotic Resistance Genes in a Rural Wastewater Treatment Plant: A Microbial Perspective of Nature-Based Versus Advanced Technologies

**DOI:** 10.3390/microorganisms13122663

**Published:** 2025-11-24

**Authors:** Lena Brouwir, Hetty KleinJan, Charlotte Balent, Gilles Quabron, Irene Salmerón, Silvia Venditti, Fanny Gritten

**Affiliations:** 1 Research and Expertise Centre for Water (CEBEDEAU), 4000 Liège, Belgium; lbrouwir@cebedeau.be (L.B.); hkleinjan@cebedeau.be (H.K.); cbalent@cebedeau.be (C.B.); gquabron@cebedeau.be (G.Q.); 2 Department of Engineering, University of Luxembourg, Campus Kirchberg rue Richard Coudenhove-Kalergi, 6, L-1359 Luxembourg, Luxembourg; irene.salmeron@ext.uni.lu (I.S.); silvia.venditti@uni.lu (S.V.)

**Keywords:** antibiotics, antimicrobial resistance, antibiotic resistance genes, constructed wetlands, ozonation, granular activated carbon, quaternary treatment, sustainable treatment technologies

## Abstract

Antibiotics (ATBs), antibiotic-resistant bacteria (ARB), and antibiotic resistance genes (ARGs) present an increasing threat/challenge to our environment and human health, resulting in increasingly strict wastewater management regulations through the revised Urban Wastewater Treatment Directive (UWWTD, 2024/3019/EU). This study evaluates a vertical-flow constructed wetland (CW) as a nature-based solution for removing ATBs, ARB, and ARGs from urban wastewater. The CW operated alongside two conventional quaternary treatments: granular activated carbon (GAC) and ozonation combined with GAC (O3 + GAC). Hydraulic conditions were kept stable across seasonal variations. Three antibiotics were quantified (through LC-MS/MS) in parallel to ARGs (through qPCR and metagenomics) and bacterial profiling (metabarcoding and plate counts). Results indicate that under the conditions tested (rural environment; UWWTP 13.000 p.e.), the CW achieves effective and stable removal of ATBs and ARGs. This study highlights the potential of nature-based solutions to match advanced quaternary treatments in removal performance and operational reliability, offering a sustainable and cost-effective means to reduce the spread of ATBs and ARGs via wastewater.

## 1. Introduction

Since the start of the 21st century, there has been a marked increase in the quality improvement of our water resources by the European Union (EU). This is evident in the various legislative measures that have been implemented or have been developed since. The Water Frame Directive (WFD) is the master legislation on surface water and groundwater protection in Europe. The objective of the WFD is to remove pollution from water bodies (quality status) to support wildlife and human needs (directive 2000/60/EC). To achieve the quality objective, the Environmental Quality Standard (EQS) directive (2008/105/EC) defines a maximum acceptable concentration of priority pollutants in the environment to avoid environmental risks [1,2] but also to protect from excessive abstraction (quantity status) [3]. To achieve the quality objective, the sister Environmental Quality Standard (EQS) directive (2008/105/EC) defines a maximum acceptable concentration of priority pollutants in the environment to avoid environmental risks [1,2]. Excessive annual average, or excessive maximum concentration in water, will force member states to improve the effluents quality of WWTP, industry and agricultural. As a precautionary measure, the EU initiated a ‘’Watch List’’ initiative in 2015 to better identify, prioritise, and monitor emerging pollutants which are suspected to pose environmental harm to surface water; assess their toxicity; and decide whether an EQS should be set for them (decision 2015/495) [4]. The first Watch List (EU 2015/495) included six pharmaceutical substances: the contraceptive 17α-ethinylestradiol (EE2); the hormone 17β-estradiol (E2); the nonsteroidal anti-inflammatory drug diclofenac; and the macrolide antibiotics erythromycin, clarithromycin, and azithromycin [4]. Indicative maximum concentration values were proposed based on conservative Predicted No-Effect Concentrations (PNECs) for direct toxicity to freshwater organisms. The antibiotics erythromycin, clarithromycin, and azithromycin were indicated with PNEC values of 0.2 µg/L, 0.13 µg/L, and 0.09 µg/L, respectively, in alignment with state-of-the-art research from the Swiss EcoTox Center [5]. This strongly suggests the high relevance of these substances for achieving good quality status; direct human toxicity is not evident, but their presence may drive resistance in pathogens. Four more Watch Lists were released up to 2025, including more drugs, antibiotics, pesticides, and UV filters.

In 2024, the recast of the Urban Wastewater Treatment Directive (UWWTD-2024/3019/EU) was published [6]. The UWWTD obligates municipalities to collect and treat pollutants (cities above 1000 population equivalent, p.e.), imposing stricter obligations for nitrogen and phosphorus removal (municipalities > 10,000 p.e., or if included in the eutrophication risk area list; Table S1). The revised UWWTD addresses the issue of micropollutants by imposing the implementation of quaternary treatments (such as oxidation and adsorption) for (1) wastewater treatment plants (WWTPs) above 150,000 p.e. capacity and (2) agglomerations larger than 10,000 p.e. if a risk for human health or the environment has been established by the member state. Compounds included in the directive are however selected differently compared to the WFD, EQS directive, and Watch Lists. They are indicators or representative substances for removal efficiencies and are therefore used as parameters designed for quaternary treatment. Urban wastewater treatment plants (UWWTPs) are widely recognised as critical hot spots for the release of a broad range of contaminants into the aquatic environment, including micropollutants—pharmaceuticals, personal care products, pesticides, and industrial chemicals—as well as antibiotics, antibiotic-resistant bacteria (ARB), and antibiotic-resistance genes (ARGs) [7,8,9,10]. Numerous studies have demonstrated that conventional WWTP processes often achieve only partial removal of these contaminants, allowing both parent substances and their transformation by-products to persist through treatment and enter receiving waters [11,12,13].

The consequences of improper removal of micropollutants are numerous, especially in low-flow water bodies [3], and their negative impacts affect numerous areas, including ecosystems [13,14], human health (e.g., antimicrobial resistance [15] and contaminated drinking water [16]), environmental safety (e.g., through bioaccumulation [17]), agriculture (e.g., via irrigation with contamination water [18,19,20]), and economics (water treatment costs [21]).

The difficulty in the selection of priority ATBs arises from the fact that the impact of antibiotics is governed not only by their concentration and persistency, but also by their intrinsic toxicity to aquatic ecosystems. The weighted average risk quotient has been proposed by Yang et al. [22], which integrates the risk intensity and frequency of micropollutants in global aquatic environments. The weakness of this quotient is that it does not consider ATB by-products, microplastics, nor the effect on antimicrobial resistance (AMR). In addition, cocktail effects may occur, with intricate interactions and the potential formation of new, occasionally more harmful by-products [23].

AMR arises when bacteria (and/or viruses, fungi, or parasites) no longer respond to antimicrobial agents, rendering standard treatments ineffective. It arises either through the acquisition of resistance genes (through horizontal transmission) or via mutations within the bacterial genome followed by vertical transmission [24]. Spread of ARGs can be facilitated by integrons, which are mobile genetic elements often associated with plasmids or transposons, enabling bacteria to capture, integrate, and subsequently express ARGs. The relative amount of ARGs from clinical isolates has been monitored by the European Centre for Disease Prevention and Control since 2010, showing an EU-wide trend of increasing ARG levels since then [25]. ARGs can be shared among bacteria, facilitating the spread of antibiotic resistance [26]. AMR has been estimated to have directly caused 1.27 million deaths, while contributing indirectly to a further 4.95 million deaths in 2019 [16,27].

Individuals are exposed to antibiotic resistance problems when the treatment of severe infections becomes compromised due to the presence of ARB, resulting in precarious, costly, and sometimes unsuccessful outcomes [28]. For example, strains of *Neisseria gonorrhoeae* have shown triple resistance to common antibiotics such as penicillins, tetracyclines, and fluoroquinolones [29,30]. Because of the need for single-dose therapy, a parenteral cephalosporin (i.e., cefixime) has remained the only available treatment option. However, a study reported decreased susceptibility of *N. gonorrhoeae* strains to cefixime, indicating the emergence of higher-level resistance and suggesting the need for agents with novel mechanisms of action [31].

This health issue has been taken up by the healthcare sector with the World Health Organization Access, Watch, and Reserve classification of antibiotics (AWaRe). Through this classification they promote the use of specific antibiotics for first- and second-line therapies (‘Access’), broad spectrum antibiotics for severe infections (‘Watch’), and finally last-resort antibiotics (‘Reserve’), aiming to mitigate further propagation of multidrug-resistance organisms in the environment. Antibiotic consumption per inhabitant in Europe has stabilised (2019–2023), with the proportion of the different antibiotic classes showing little change (penicillins, macrolides, β–lactams, tetracyclines, quinolones, sulfonamides, and others from minor groups combined). However, the proportion of reserve antibiotics consumed out of the hospital has risen [16].

Integrating environmental considerations into antibiotics use is a challenging process; therefore the UWWTD is essential for establishing a primary barrier to the dissemination of micropollutants, ARB, and ARGs into the environment. The monitoring requirements imposed by UWWTD are essential for establishing a baseline for the micropollutants present in treated waters, and to assess the performance of (novel) treatments. The identification and measurement of micropollutants, therefore ATBs, require advanced and highly sensitive analytical methods (e.g., LC-MS/MS). These methods require instruments that demand substantial financial investment while also needing highly specialised personnel. In addition, each target compound typically requires the development of a custom analytical protocol. As an alternative, methods based on molecular biology are targeting the genetic components involved in micropollutant toxicity, degradation, transfer, or resistance, rather than the micropollutant directly.

The detection of ARB and their ARGs can be achieved through culture-based and molecular methods. Culture approaches, adapted from clinical microbiology, use membrane filtration and selective media followed by antibiograms [32]. They are robust, low-cost, and provide direct evidence of resistance, but remain labour-intensive, time-consuming, and cannot detect viable but non-culturable bacteria or capture the full microbial diversity present in environmental samples [33]. Molecular techniques, including quantitative polymerase chain reaction (qPCR) [10], DNA sequencing, metabarcoding, and metagenomics, enable detection and quantification of ARGs after DNA extraction and can be applied to both clinical isolates and complex environmental communities [34]. Their main limitations include the influence of PCR inhibitors, the detection of unwanted extracellular or dead-cell DNA, the lack of standardised protocols, and higher analytical costs [35,36].

To comply with the UWWTD requirements, municipalities are exploring quaternary treatments such as advanced oxidation processes, granular activated carbon, and nature-based solutions, such as constructed wetlands. These are particularly attractive as lower-cost, easier-to-operate alternatives to more advanced technologies and have demonstrated potential for micropollutant removal [37,38,39,40,41].

This study responds to the environmental challenges of minimising antibiotic discharge from urban wastewater by evaluating the performance of a nature-based solution for the removal of antibiotics, ARGs, and ARB from the effluent of a WWTP located in Bliesen (Germany). Specifically, the concentrations of two antibiotics and one metabolite are reported, alongside the detection of ARGs via qPCR and metagenomics, and ARB through long-read bacterial metabarcoding. Although ARGs are not yet regulated, this monitoring follows emerging guidelines currently under development by Schwermer et al. [10]. Practically, a vertical-flow constructed wetland was operated for one year in parallel with two conventional quaternary treatments: (1) granular activated carbon and (2) a combination of ozonation and granular activated carbon. Hydraulic conditions were kept stable over the experiment to collect sufficient data for evaluating treatments’ performances over time and across seasonal variations under both dry- and wet-weather conditions.

## 2. Materials and Methods

### 2.1. Wastewater Treatment Plant and Experimentation

**Figure 1:**
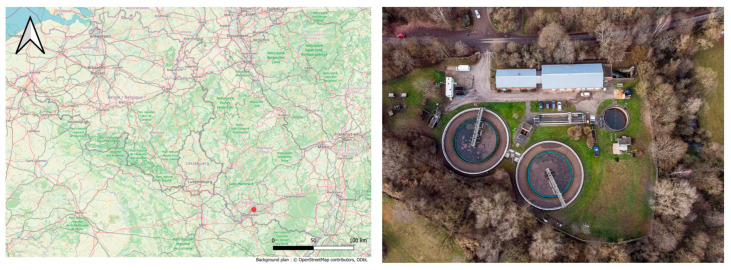
(**Left**) Location of the pilot plant in Germany. The arrow indicates north, and the red dot is the location of the wastewater treatment plant in Bliesen. (**Right**) Aerial view of the treatment plant of Bliesen.

The pilot plant was installed in the WWTP of Bliesen (49°29′38″ north, 7°06′50″ east) in the east of Germany (Figure 1—left). The WWTP (Figure 1—right) treatment was composed of a primary treatment (oil, grease, and suspended solids) followed by a conventional activated sludge system (anoxic/oxic for total nitrogen removal) combined with chemical addition for phosphorus removal (FeCl_3_). The facility treated sewage from 13,000 inhabitants, including industrial wastewater from a bakery. The WWTP discharged the treated water into the river Blies (main tributary of the Sarre river with an annual main flowrate of 18.2 m^3^/s). 

Three technologies were tested for the quaternary treatment of ATBs, ARB, and ARGs: constructed wetland (CW), adsorption with granular activated carbon (GAC), and the combination of ozonation and granular activated carbon adsorption (O3 + GAC). The pilots treat the wastewater secondary effluent and performed the treatment for a period of one year from November 2021 to October 2022. Details of the operation are found in Salmerón et al., 2025; however a brief description is given below [42].

The WWTP outlet was continuously pumped from the water sump to a buffer tank from which the water was sent to the different treatment lines. To reduce the solids in the WWTP effluent, a filtration unit was added to the buffer tank at the beginning of October 2022 (model 2” Compact Air-Aided Flushing from Amiad Water Systems Ltd., Amiad, Israel). 

**Figure 2:**
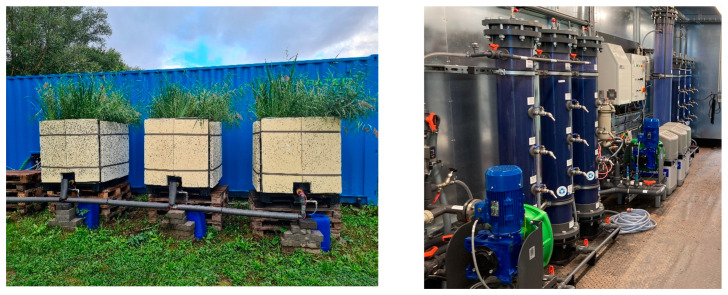
(**Left**) Constructed wetland (CW) pilot in summer 2022. (**Right**) Three columns in series of granular activated carbon (GAC) in the foreground and reactive ozone column followed by three columns in series of granular activated carbon (O3 + GAC) in the background.

The CW treatment line (Figure 2—left) consisted of a 1 m^3^ tank with a 65 cm high substrate composed of 85% sand (0.3 mm) and 15% biologically activated biochar in pellet form mixed homogeneously. This main substrate was protected by two layers of gravel, one at the bottom (10 cm of 4 to 10 mm gravel + 5 cm of 2 to 5 mm) and the other at the top (10 cm of 4 to 10 mm expanded clay), reaching a final height of 90 cm. The unit was planted with 15 plants each of common macrophytes *Phragmites australis* and *Iris pseudacorusm*, distributed alternately. The CW was watered 3 times per day with a maximum Hydraulic Loading Rate (HLR) of 400 L day^−1^ m^−2^ [42,43].

The GAC treatment line (Figure 2—right) consisted of three activated carbon columns (CGF 8×30/85 from CarboTech, Essen, Germany) operated in series with a total volume of 221 L. The filling ratio was 83%. The pilot was operated at constant flow, leading to an ascensional speed of 3.7 m/h and total empty bed contact time (EBCT) of 61 min (20 min EBTC per column). The pilot reached a bed volume of 5381 at the end of the experiment.

The O3 + GAC treatment line was composed of an ozonation system (Topaz PSA oxygen generator and Modular HC4 from WEDECO by xylem, Washington, DC, USA) followed by granular activated carbon adsorption. The ozone concentration in the water was fixed to 0.010 g O_3_/L with an average of 0.69 +/− 0.12 g O_3_/g COD during the entire experiment. The reaction tank had a volume of 103 litres resulting in a contact time of 34 min. The GAC column was operated like the GAC line alone. The unit reached a bed volume of 4584 at the end of the experiment.

### 2.2. Sample Collection and Analysis

Samples were collected before the WWTP (IN WWTP); after the WWTP (OUT WWTP); and after the CW, GAC, and O3 + GAC quaternary treatments. A delay of 24 h was applied between the sample starting at the WWTP’s inlet and the other samples to consider the hydraulic retention time in the WWTP. Thirteen campaigns were carried out, with four intensive campaigns with a 72 h sampling period, and nine light campaigns with a 24 h sampling period, both based on composite samples. Grab samples were taken for the CW at the end of the sampling period of the other lines (Table S3). Flow proportional autosamplers were used at the WWTP inlet and outlet while time-proportional autosamplers were used for the collection of GAC and O3 + GAC effluents. The total volume sampled was split to ensure physicochemical (macropollutant), ATB, and microbial analysis.

### 2.3. Monitoring of Physicochemical Parameters

The main physical characteristics of the WWTP effluent, such as turbidity, pH, conductivity, and redox potential, were monitored continuously by in-line probes connected to a central programmable logic controller. The carbon content was measured with the total organic carbon (TOC) following NF EN 1484 using TOC-VCPN (Shimadzu, Tokyo, Japan). The chemical oxidation demand (COD) was measured using kits (LCI 500 according to ISO 15705 or LCK1014 according to ISO 6060-1989 from Hach Lange, Düsseldorf, Germany). The biological oxygen demand measured after five days (BOD_5_) was measured using the WTW™ OxiTop™-i Respirometric BOD Measurement System following NBN EN 1899-1 (1998) (fisher scientific, Waltham, MA, USA). Ammonium (NH_4_), nitrate (NO_3_), and nitrite (NO_2_) were analysed by continuous flow analysis according to ISO 11732 (NH_4_) and ISO 13395 (NO_3_ and NO_2_). Total phosphorus concentration was measured following NF EN ISO 6878 using a UV-visible Shimadzu spectrometer (Shimadzu, Tokyo, Japan). Total suspended solid (TSS) was analysed according to the NF EN 872 norm. The volatile suspended solid was calculated with the following formula: (TSS_105 °C_ − TSS_525 °C_)/TSS_105 °C_.

### 2.4. Monitoring of Antibiotics Levels

Liquid Chromatography coupled to tandem Mass Spectrometry was used to measure the concentration of antibiotics (Table S4). It consisted of an Agilent 1200 SL LC (Agilent Technologies, Santa Clara, CA, USA) coupled with a Hybrid Quadrupole-Linear Ion Trap instrument (4500 QTrap from Sciex, Framingham, MA, USA) with electrospray ionisation in positive mode operated in Multiple Reaction Monitoring. All samples were pre-concentrated by solid phase extraction before their injection. In this study, the impact of quaternary treatments on the removal of antibiotics was studied for clarithromycin (CLA), sulfamethoxazole (SMX), and the metabolite of SMX, N-Acetylsulfamethoxazole (N-SMX). The antibiotics selected are relevant in the framework of discharge requirements for urban wastewater treatment plants (clarithromycin is a First Category micropollutant, UWWTD-2024/3019/EU; Table S2), and/or are listed in the third Watch List (sulfamethoxazole and N-Acetylsulfamethoxazole; 2008/105/EC [2], EU Commission Decision 2020/1161 [44], and Gomez et al., 2020 [45]). They were considered relevant for the Bliese catchment because they were excreted in high amounts.

### 2.5. Monitoring of Microbial Parameters

#### 2.5.1. Quantification of Antibiotic Resistance, Class 1 Integrase, and 16S rRNA Genes

A range of antibiotic resistance genes was selected based on research conducted in 2022 and are in line with the recommendations of the European Environment Information and Observation Network (EIONET) working group [10] published in 2025. Six key genes were identified according to specific criteria, including their frequent occurrence, ease of detection despite low abundance, clinical relevance, capacity to reflect human exposure, and suitability for assessing the effectiveness of AMR mitigation measures. Three additional genes were considered optional for detection. In the present study, six out of the nine recommended genes were selected [46,47,48]: 16S rRNA gene, used as a proxy for total bacterial abundance; *intI1*, the class 1 integrase gene associated with mobile genetic elements; *blaAmpC* (substituted for *bla_CTX-M1_*), linked to β-lactamase resistance; *ermB*, conferring resistance to macrolides; *tetW*, associated with tetracycline resistance; and *sul1*, indicating resistance to sulfonamides.

Analysis via qPCR was carried out in the influents and the effluents after the different treatments. Between 100 and 2500 mL of wastewater (Table S5) was filtered through PES membranes (0.1 µm, Pall Corporation, Port Washington, NY, USA) and subsequently subjected to DNA extraction using the proprietary protocol, with an elution volume ranging from 20 to 90 µL (Table S6) (DNeasy^®^ PowerWater^®^ Kit; Qiagen N.V., Venlo, The Netherlands). After quantification (NanoDrop™ One, Thermo Fischer Scientific™, Waltham, MA, USA; Table S7), 3 ng of DNA was used as input material for qPCR analyses. The qPCR reactions were performed in a final volume of 20 µL, which consisted of 0.6 µL of 10 µM forward primer, 0.6 µL of 10 µM reverse primer, 10 µL of 2× GoTaq^®^ qPCR SYBR Green, 3 µL of 1 ng/µL DNA, and 5.8 µL of nuclease-free water (Promega, Madison, WI, USA). The primers used and the thermal reaction procedure for each gene are referenced in Table 1. Melting curves (5 s/cycle, 0.5 °C/cycle) were analysed by comparing the melting temperature of the standard with that of the samples to discriminate any non-specific amplification (Protocol S-1), which were run in parallel. Each sample was analysed using four technical replicates. ARG concentrations were finally normalised to the concentration of the 16S rRNA gene.

#### 2.5.2. Culture-Based Enumeration of Indicator Bacteria

Plate counts were acquired using standardised methods for the following: (i) total aerobic bacteria at 22 °C and (ii) at 36 °C (CFU/mL; NBN ISO 6222); (iii) coliform bacteria (CFU/100 mL; BRD 07/20-03/11); (iv) faecal coliforms (CFU/100 mL; method derived from BRD 07/20-03/11); (v) *Escherichia coli* (CFU/100 mL; BRD 07/20-03/11); and (vi) intestinal *Enterococci* (CFU/100 mL; NBN ISO 7899-2). Analyses were outsourced to the CILE (Compagnie Intercomunnale Liégeoise des Eaux, Liège, Belgium) laboratory.

#### 2.5.3. Flow Cytometric Enumeration of Total and Intact Bacteria

Total cell counts (TCCs) of the water samples were measured according to CEBEDEAU’s in-house analytic protocol adapted from [49]. After resuspending the water sample (manual shaking), 0.5 mL of water was incubated (15 min, 37 °C) with 1X SYBR^TM^ Green I Nucleic Acid Gel Stain (Invitrogen ^TM^ by Thermo Fischer Scientific™, USA). For the determination of the percentage of intact cell counts (ICCs), sample preparation was identical, except that 0.003 mM of Propidium Iodide (Invitrogen ^TM^ by Thermo Fischer Scientific™, USA) was added in addition to the SYBR™ Green. Quantification was performed using the BD Accuri ^TM^ C6 Plus flow cytometer (Becton, Dickinson and Company, Franklin Lakes, NJ, USA) in its standard configuration (3 blue and 1 red laser, optical filters of 533/30 nm (FL1), 585/40 nm (FL2), 670 LP nm (FL3), and 675/25 nm (FL4)), with the proprietary BD Accuri C6 Plus software (version 1.0.34.1). Data acquisition was carried out using the following configuration: run time 30 s., flow rate 14 µL/min, and a primary threshold of FL1-H 800. Quantification was accomplished through the signal captured by the FL1 and FL3 detectors, implementing standardised gating.

#### 2.5.4. Bacterial Community Profiling with Metabarcoding

The nanopore protocol for full-length 16S rRNA gene sequencing (version 16_v1_revM_14Aug2019, sequencing kit: SQK-16S024) was used as a reference method, using 10 ng of extracted DNA for the DNA fragment specific amplification. An equimolar pool of barcoded libraries was prepared in 10 µL buffer (10 mM Tris-HCl pH 8.0 with 50 mM NaCl), corresponding to 50–100 fmoles, which equates to ~50–100 ng of PCR product in total (~1–10 ng per sample). The final library was sequenced using a MinION flow cell (Spot-on flow cell, R9 R9.4.1 chemistry, model FLO-min106; Oxford Nanopore Technologies, Oxford, UK). Sequencing data was generated using MinKNOW v24.06.10 software Oxford Nanopore Technologies, Oxford, UK). Reads were live-basecalled with the basecalling algorithm v4.3.0 from Dorado V7.4.12 Oxford Nanopore Technologies, Oxford, UK). Global quality of the sequencing was evaluated with Nanoplot [50], while quality filtering was set on a Q score threshold of 10. Reads were length-filtered (1400 to 2000 bp) using Filtlong [51]. A total of approximately 10,000 reads were generated for each sample (except for 4 out of 16 samples).

Taxonomic assignment (to species level) was conducted using EMU v3.5.0 algorithm [52] and EMU database v3.4.5. Community composition and alpha diversity analyses were performed with R v4.2.3 using Phyloseq v1.42.0 [53]. Plots were then generated using ggplot2 v3.5.1 [54]. Beta diversity was assessed by non-metric multidimensional scaling (NMDS) using MicrobiomeAnalyst v2.0 [55]. Prior to analysis, reads were filtered to reduce low counts (minimum of 4 counts) and low variance features (10% inter-quartile range). Differences between samples were evaluated using PERMANOVA analysis included in MicrobiomeAnalyst.

#### 2.5.5. Antibiotic Resistance Gene Profiling with Metagenomics

In-house sequencing of the CW sample and OUT WWTP sample from campaign 4 (C12-I) was performed on the MinION Mk1B device (ONT, UK) using the Rapid Barcoding Kit 24 V14 (SQK-RBK114-24, Oxford Nanopore Technologies, Oxford, UK) according to the manufacturer’s instructions. Sequencing data were generated using MinKNOW v24.06.10 (ONT, UK). Reads were live-basecalled with the super-accurate basecalling algorithm v4.3.0 from Dorado v7.4.12 (Oxford Nanopore Technologies, Oxford, UK). The quality of the reads was evaluated with Nanoplot [50].

Reads were assembled into contigs by Flye v2.9.5-b1801 [56]. The resulting assembled contigs were then indexed and mapped with minimap2 v2.28-r1209 [57], sorted with Sambamba v1.0.1 [58], and binned with MetaBAT v2.15 [59], resulting in metagenome-assembled genomes (MAGs). GENERA toolbox [60] allowed assessment of MAGs’ quality with CheckM v1.2.2 [61] and MAGs’ taxonomy identification with GTDB-tk v2.0.0 [62]. From the assembled contigs, antimicrobial genes were identified using the AMRFinder plus tool [63].

### 2.6. Data Treatment

#### 2.6.1. Macropollutants and Antibiotics

Nutrient and ATB analysis results below the limit of quantification (LOQ) were set to the LOQ as a conservative approach to evaluate treatment performance. Removal performance was calculated for the WWTP after tertiary treatment (R_WWTP_, Equation (1)) and for the total treatment, including the WWTP and the quaternary treatment R_WWTP+X_ (X = CW, GAC or O3 + GAC; Equation (2)). The difference between R_WWTP_ and R_WWTP+X_ corresponded to the contribution of the quaternary treatment (R_CW_, R_GAC_, or R_O3 + GAC_; Equation (3)).

(1) $$\mathrm{Removal}\;\mathrm{WWTP}\;(R_{\mathrm{WWTP}},\;\%)=(1-\frac{C_{E}}{C_{I}})\times 100$$

(2) $$\mathrm{Removal}\;\mathrm{WWTP}+\mathrm{quaternary}\;\mathrm{treatment}\;(R_{\mathrm{WWTP}+X},\;\%)=(1-\frac{C_{x}}{C_{I}})\times 100$$

Removal quaternary treatment (R_CW_, R_GAC_ or R_O3+GAC_, %) = R_WWTP+X_ − R_WWTP_ (3)

Within the equations, C_E_ is the concentration of the WWTP effluent; C_I_ is the concentration of the WWTP influent; C_X_ is the concentration at the effluent of the quaternary treatment line; and R_X_ corresponds to the removal performance of a quaternary treatment (for both X = CW, GAC, or O3 + GAC). Positive removals correspond to a reduction in the pollutant in the treated water (relative to non-treated influent water), while negative removals correspond to a discharge/release of the pollutant relative to the non-treated influent water.

#### 2.6.2. Microbial Parameters

Each result from ARG and cytometry was expressed as a percentage reduction relative to the sample taken as the reference sample. In addition, log reduction (L) values were calculated according to Equation (4):

(4) $$L=log\left(N_{0}\right)-log\left(N_{t}\right)$$

where $N_{0}$ is the concentration in the reference sample and $N_{t}$ is the concentration after the treatment under study.

The percentage reduction (P) was calculated according to Equation (5):

(5) $$P=\left(1-10^{-L}\right)\times 100$$

A positive percentage indicates a reduction in concentration, whereas a negative value reflects an increase compared to the reference sample. For ARGs, absolute concentrations (expressed in gene copies/L) were normalised to the concentration of the 16S rRNA gene, providing the relative abundance of ARGs and the class 1 integrase gene expressed as gene copies per 16S rRNA gene copy. Absolute concentrations and relative abundances were calculated as the means of four technical replicates.

Plots were generated with R v4.2.3 using tidyverse v2.0.0 [64] and ggplot2 v3.5.1 [54].

## 3. Results

Results are presented from thirteen monitoring campaigns: nine light campaigns (L) and four intensive campaigns (I). The light campaigns included analyses of macropollutants and antibiotics, while the intensives ones included the analyses of the ARGs by qPCR and metagenomics, bacterial community profiling by long-read metabarcoding, cell counts by flow cytometry, as well as macropollutants and antibiotics. Sampling points and treatment lines are denoted as follows: WWTP influent (IN WWTP), WWTP effluent (OUT WWTP), constructed wetland (CW), granular activated carbon (GAC), and ozonation followed by GAC (O3 + GAC).

Information about campaigns (ID, weather condition, buffer tank physicochemical parameters) can be found in Table S3 and are summarised in Table 2. During the experimental study, performances were evaluated during the four seasons considering both dry- and wet-weather conditions. The CW pilot was installed in November; for this reason, during the first months of operation, adsorption is expected to be the main degradation pathway of nutrients and ATBs, as plants need an adaptation period that is longer due to the winter conditions. At the end of the experiment (autumn), all degradation pathways should occur inside the constructed wetland: biodegradation, adsorption and plant uptake [23].

### 3.1. Impact of Treatment on Nutrients

During the experiment, the WWTP received an average flow of 4 253 m^3^ per day and discharged a treated effluent of 18 mg COD/L; <5 mg BOD_5_/L; 4 mg TSS/L; 0.7 mg N-NH_4_/L; 2.1 mgN-NO_3_/L; and 0.5 mg P/L, which gave a COD/N/P ratio at the inlet of the quaternary treatment of 100/3.9/2.7. No significant changes were shown after the CW for COD, whereas nitrification and phosphorus removal increased by 3% and 10%, respectively. In GAC and O3 + GAC, the ammonium, COD, and P concentrations did not show significant changes compared to the secondary treatment effluent. Effluents from all quaternary treatments complied with the UWWTD requirements (Tables S1 and S8).

### 3.2. Impact of Conventional Activated Sludge Treatment on the Occurrence and Distribution of Antibiotics and Antibiotic Resistance Genes in Treated Wastewater

#### 3.2.1. Antibiotics

**Figure 3:**
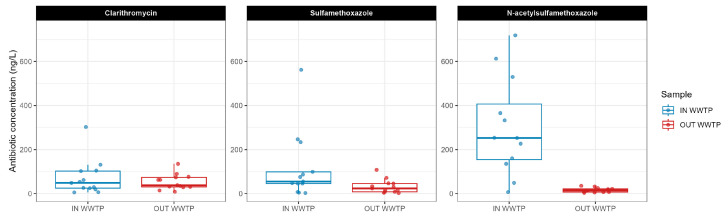
Concentration of antibiotics (clarithromycin, sulfamethoxazole, N-acetylsulfamethoxazole) measured in the influent (IN WWTP) and effluent (OUT WWTP) during thirteen sampling campaigns. Data are expressed in ng/L. One measurement was masked (IN WWTP, N-acetylsulfamethoxazole of 1453.46 ng/L for a better figure visualisation).

The presence of three antibiotics (CLA, SMX, and N-SMX) in the influent and effluent of the wastewater treatment plant was investigated. The concentrations of CLA, SMX, and N-SMX were above the LOQ in all samples throughout all thirteen sampling campaigns—except for four (CLA in C1 and C3 for IN WWTP sample; SMX in C13 for IN and OUT WWTP samples; Table S9). Antibiotic concentrations in IN WWTP samples varied between 2.7 and 1453 ng/L and in OUT WWTP samples varied between 2.5 and 135.4 ng/L across all campaigns (Figure 3).

**Figure 4:**
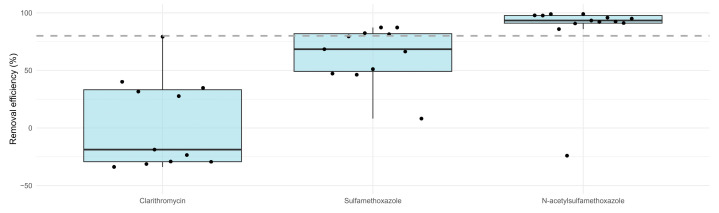
WWTP removal efficiency calculated for three antibiotics (clarithromycin, sulfamethoxazole, and N-acetylsulfamethoxazole) for thirteen campaigns according to Equation (1). Data are expressed as percentage reduction in the antibiotic concentrations measured in the influent compared to the effluent. Four values below −50% were masked for better figure visualisation. The dots represent the removal efficiency of each campaign. The dashed grey line indicates 80% removal efficiency.

Antibiotic removal by the WWTP is reported in Figure 4. The WWTP achieved more than 80% reduction in N-SMX for 12 out of the 13 sampling campaigns. SMX median removal efficiency was 66.3%. In contrast, several percentage removals of CLA presented a negative value, (median reduction of −23.5%), demonstrating the poor removal of this molecule by the WWTP alone.

#### 3.2.2. Antibiotic Resistance Genes and Class 1 Integrase Gene

The presence of antibiotic resistance genes and the class 1 integrase gene in the influent and effluent of the WWTP (without quaternary treatments) was investigated. Two campaigns evaluated the concentrations in the IN WWTP and four campaigns for the OUT WWTP, with four technical replicates each. The data show that the gene copy concentrations (Table S10), based on data not normalised by 16S rRNA gene concentrations, in the IN WWTP samples varied between 1.97 × 10^11^ and 1.37 × 10^13^ gene copies/L and that gene copy concentrations in OUT WWTP samples varied between 7.16 × 10^7^ and 8.12 × 10^10^ gene copies/L. The most abundant genes detected in both IN WWTP and OUT WWTP were the *intI1*, *ermB*, and *sul1* followed by *tetW* and *blaAmpC*. The *intI1* concentrations in IN WWTP samples varied between 2.82 × 10^12^ and 1.31 × 10^13^ gene copies/L and in OUT WWTP samples between 1.41 × 10^10^ and 8.12 × 10^10^ gene copies/L. The 16S rRNA gene concentrations for both IN WWTP and OUT WWTP were above the concentrations of all other genes measured (IN WWTP: between 8.39 × 10^13^ and 1.98 × 10^14^ copies/L; OUT WWTP: between 1.97 × 10^11^ and 1.99 × 10^12^ copies/L).

**Figure 5:**
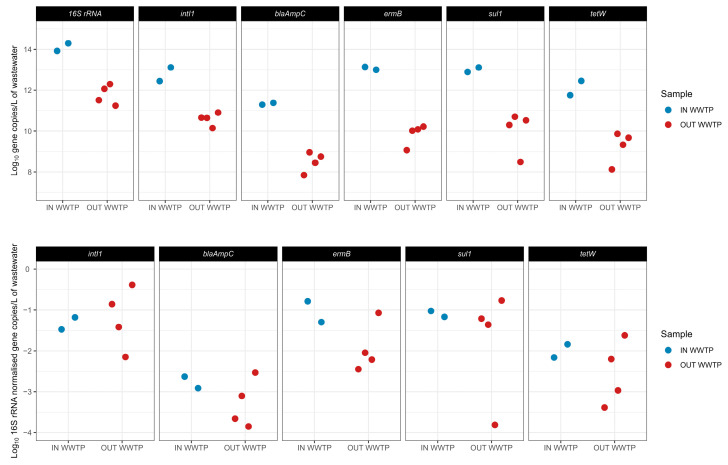
Concentrations of antibiotic resistance genes *(blaAmpC*, *ermB*, *sul1*, and *tetW*) and class 1 integrase gene (*intI1*) measured in the WWTP influent (IN WWTP) and effluent (OUT WWTP); each dot represents one campaign and shows the mean value of four technical replicates. (**Upper panel**): visualisation of log-transformed data; (**Lower panel**): visualisation of log-transformed concentrations normalised by 16S rRNA gene concentrations (relative abundances).

The data indicate that the prevalence of ARGs and class 1 integrase gene concentrations expressed as gene copies relative to the gene copies of the 16S rRNA gene in IN WWTP samples varied between 2.35 × 10^−3^ and 6.79 × 10^−2^ and ARG concentrations in OUT WWTP varied between 1.41 × 10^−4^ and 4.11 × 10^−1^ (Figure 5). The most abundant genes detected based on the normalised data were the same as the non-normalised data. The *intI1* concentrations varied between 3.36 × 10^−2^ and 6.60 × 10^−2^ in IN WWTP samples and between 7.07 × 10^−3^ and 4.11 × 10^−1^ in OUT WWTP samples.

**Figure 6:**
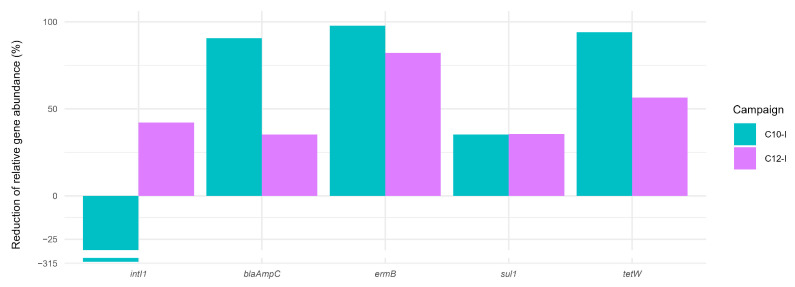
WWTP removal efficiency calculated for antibiotic resistance genes (*blaAmpC*, *ermB*, *sul1*, and *tetW*) and class 1 integrase gene (*intI1*) according to Equation (5). Data are expressed as percentage reduction in the gene concentrations (data normalised by 16S rRNA gene concentrations) measured in the influent compared to the effluent.

ARG and class 1 integrase gene removal rates were calculated to assess the capacity of the WWTP to eliminate them at C10-I and C12-I. For the four ARGs analysed (*blaAmpC*, *ermB*, *sul1*, and *tetW*), removal efficiencies ranged from 35.17% (*blaAmpC*, C12-I) to 97.82% (*ermB*, C10-I) (Figure 6). The WWTP achieved more than 80% reduction in the *ermB* gene during both campaigns. In contrast, the *intI1* concentration increased (313.99%) during the C10-I campaign, indicating a higher prevalence of gene transfers in the OUT WWTP compared to the IN WWTP. Conversely, during the C12-I campaign, *intI1* concentration was reduced (42.02%).

### 3.3. Impact of Quaternary Treatment on Occurrence and Distribution of Antibiotics, Antibiotic Resistance Genes, and Microbial Indicator Organisms in Treated Wastewater

#### 3.3.1. Antibiotics

**Figure 7:**
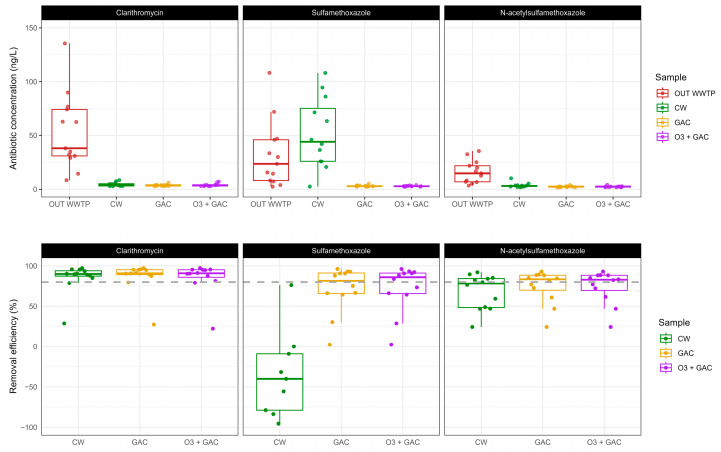
(**Upper panel**): Antibiotic concentrations (clarithromycin, sulfamethoxazole, N-acetylsulfamethoxazole) measured for each campaign in the WWTP effluent (OUT WWTP) and after quaternary treatments (CW, GAC, O3 + GAC) expressed in ng/L. (**Lower panel**): Antibiotic removal efficiency calculated according to Equation (2) and expressed as percentage reduction in concentration measured in the OUT WWTP compared to the effluent after quaternary treatment for each campaign. The dotted line represents 80% removal. Three values for CW–sulfamethoxazole are masked (−1234.87, −431.03, and −307.10%); only −1234.87% is considered an outlier (>1.5 × inter-quartile range (IQR)) and is excluded from the boxplot calculation.

The presence of three antibiotics was monitored in the effluent of the WWTP and after the three quaternary treatments (GAC, O3 + GAC, and CW) (Figure 7—upper panel). Antibiotic concentrations in the OUT WWTP varied between 2.51 and 135.44 ng/L. Concentrations after quaternary treatment were between 1.89 and 6.11 ng/L (GAC), 1.93 and 108.03 ng/L (CW), and 1.89 and 7.09 ng/L (O3 + GAC).

Antibiotic concentration after quaternary treatment was, in general, more stable (less scatter in data). CLA was the most abundant (median = 38.1 ng/L), followed by SMX (median = 23.5 ng/L) and its metabolite N-SMX (median = 14.7 ng/L).

Removal efficiencies for all three antibiotics were ≥80% (Figure 7—lower panel), except for SMX in CW, where negative values (median = −47.9%) indicated a net release of the molecule rather than a removal. This release was not observed for its metabolite N-SMX.

#### 3.3.2. Antibiotic Resistance Genes and Class 1 Integrase Gene

The presence of antibiotic resistance genes (*blaAmpC*, *ermB*, *sul1*, *tetW*) and class 1 integrase gene (*intI1*) in the effluent of the wastewater treatment plant and after additional quaternary treatment (GAC, O3 + GAC, CW) was examined. The data show that the gene copy concentrations, based on data not normalised by 16S rRNA gene concentrations (Table S10, Figure S1—upper panel), varied between 4.29 × 10^7^ and 9.73 × 10^9^ gene copies/L for GAC; 2.63 × 10^7^ and 6.18 × 10^10^ gene copies/L for O3 + GAC; and between 3.62 × 10^6^ and 6.27 × 10^7^ gene copies/L for CW. The most abundant genes detected among all treatments were *intI1* (6.18 × 10^10^ to 6.27 × 10^7^ copies/L), followed by *ermB* (1.13 × 10^1^ to 2.63 × 10^7^ copies/L) and *sul1* (2.69 × 10^10^ to 1.51 × 10^7^ copies/L). Regarding the prevalence of ARGs and the class 1 integrase gene expressed as gene copies (relative to the gene copies of the 16S rRNA gene) after quaternary treatment (Figure S1—lower panel), values varied between 4.83 × 10^−5^ and 6.23 × 10^−2^ for O3 + GAC, between 2.90 × 10^−4^ and 2.35 × 10^−2^ for GAC, and between 1.73 × 10^−4^ and 1.18 × 10^−2^ for CW. The most abundant genes detected were the same as in the non-normalised data.

**Figure 8:**
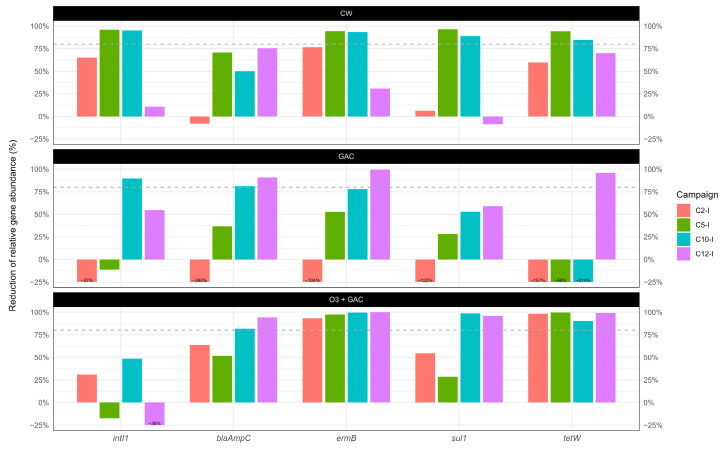
Percent reduction abundance calculated for antibiotic resistance genes (*blaAmpC*, *ermB*, *sul1*, and *tetW*) and the class 1 integrase gene (*intI1*) according to Equation (5). Data are expressed as percentage reduction in the gene concentrations measured in the WWTP effluent compared to the treatment line understudy normalised by the 16S rRNA gene concentrations. The dashed grey line indicates 80% removal efficiency.

Removal efficiencies were calculated based on Equation (5) (Figure 8). For the four ARGs analysed (*blaAmpC*, *ermB*, *sul1*, and *tetW*), removal efficiencies ranged between 99.5% (C12-I, ermB) and 6.15% (C2-1, *sul1*). Negative removal rates were observed in the GAC treatment for *tetW* (e.g., −214.4% at C10-I), *blaAmpC* (e.g., −260.04% at C2-I), *sul1* (e.g., −121.7% at C2-1), *intI1* (e.g., −37% at C2-1), and *ermB* (e.g., −104% in C2-1). Similarly, for the O3 + GAC treatment, negative removal rates for *intI1* (e.g., −38.2% at C12-1) were calculated.

#### 3.3.3. Bacterial Community Composition

Long-read 16S rRNA gene metabarcoding sequencing (Tables S11 and S12) was performed on effluent from the WWTP and after quaternary treatment (OUT WWTP, CW, GAC, and O3 + GAC) during the four intensive campaigns (C2-I, C5-I, C10-I, and C12-I).

Alpha diversity was calculated as the median across campaigns per treatment. Across all three indices selected (species richness, Shannon, and Simpson), CW consistently supported the highest microbial diversity compared to the OUT WWTP and the other quaternary treatments (Figure S2).

**Figure 9:**
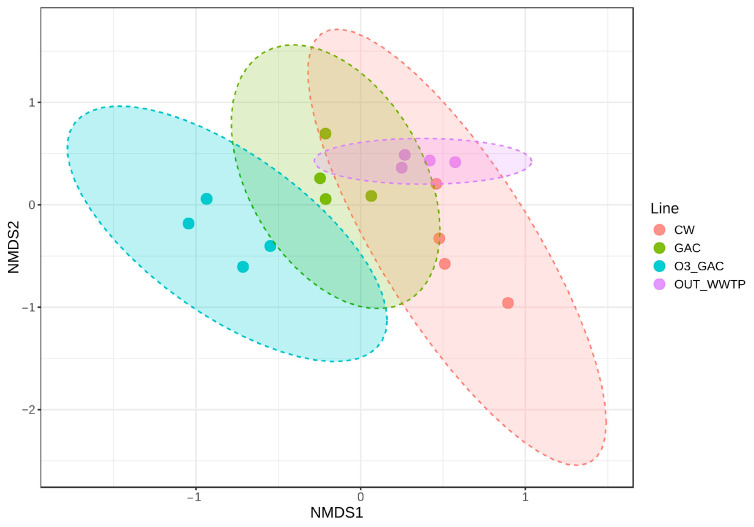
Beta diversity assessed by non-metric multidimensional scaling (NMDS) based on Bray–Curtis distances for the WWTP effluent (OUT WWTP, purple) and the quaternary treatments (CW, red; GAC, green; O3 + GAC, blue) across the four intensive campaigns (C2-I, C5-I, C10-I, and C12-I). Ellipses represent a 95% confidence interval.

Beta diversity analysis (Figure 9) indicated that the OUT WWTP communities remained very close to each other across the four intensive campaigns. These communities were not significantly different from those found in the CW (*p* = 0.063). In contrast, communities from the GAC and O3 + GAC treatments differed significantly from the OUT WWTP (*p* = 0.036 and *p* = 0.029, respectively). Moreover, the addition of ozonation in the O3 + GAC treatment induced a distinct and significant shift (*p* = 0.028) in the microbial community compared to GAC alone.

**Figure 10:**
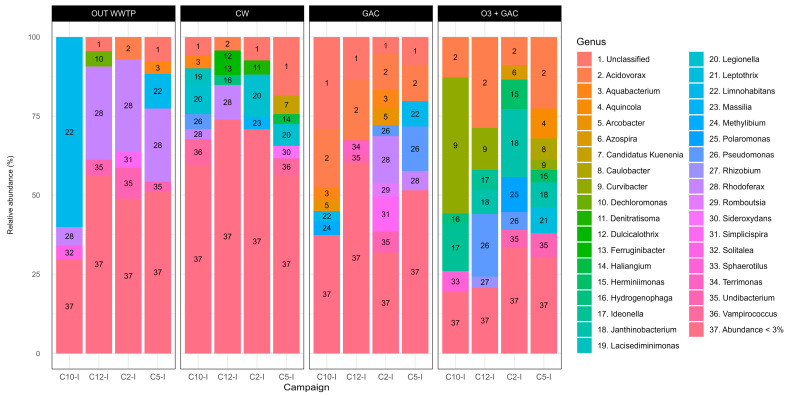
Community composition at the genus level in the WWTP effluent (OUT_WWTP) and after quaternary treatment (CW, GAC, and O3 + GAC) across the four intensive campaigns (C2-I, C5-I, C10-I, and C12-I). Genera with a relative abundance <3% are grouped together.

Variations in the community composition over the four intensive campaigns are assessed by the relative abundances at the genus level in the OUT WWTP and after quaternary treatments (genus abundances under 3% are grouped together) (Figure 10). The community in OUT WWTP is homogenous across campaigns. *Limnohabitans* and *Rhodoferax* abundances range between 1 and 10% and around 25%, respectively, across all campaigns, except for C10-I, where a shift in abundance occurs (60.10% and 5.81%, respectively). In CW, low abundance taxa (<3.0%) account for >50% of the total relative abundance in CW, compared to values <50% in other treatments. The four most abundant genera in CW are *Rhodoferax* (0–10.94%, which are, respectively, minimum and maximum abundances across all campaigns), *Denitratisoma* (0.44–4.45%), *Pseudomonas* (0.31–4.96%), and *Legionella* (1.42–13.34%). The community in the GAC shows the dominance of *Acidovorax* (11.34–19.07%). Other genera with noticeable variations are *Pseudomonas* (1.06–14.03%), *Rhodoferax* (1.26–14.88%), and *Simplicispira* (0.71–11.09%). Community in the O3 + GAC displays a broader set of dominant genera, with *Curvibacter* showing the largest variation across campaigns (0.29–43.03%), followed by *Acidovorax* (8.90–28.76%), *Pseudomonas* (1.31–19.83%), *Janthinobacterium* (0.99–21.48%), *Ideonella* (0–14.64%), *Polaromonas* (0–11.02%), *Aquincola* (0.20–9.40%), and *Herminiimonas* (0.30–9.31%).

#### 3.3.4. Indicator Bacteria

**Figure 11:**
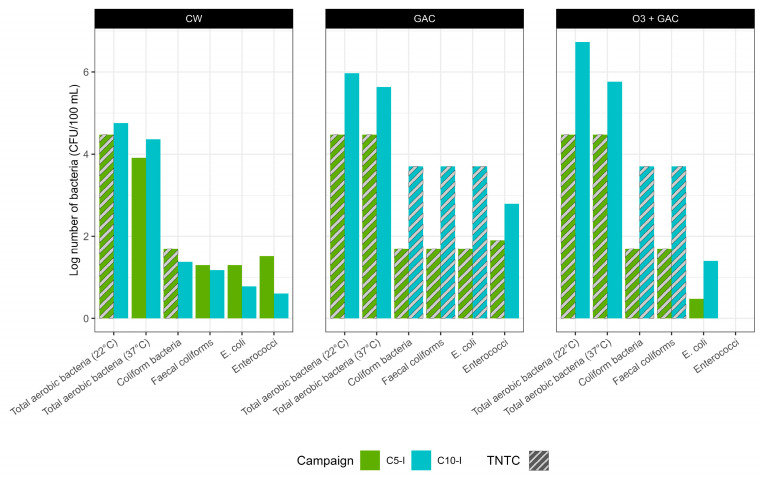
Culture-based cell counts of indicator bacteria (total aerobic bacteria at 22 °C and 37 °C, coliforms, faecal coliforms, *Escherichia coli*, and *Enterococci*) are measured after quaternary treatment during C2-I and C10-I. Data are expressed in CFU/100 mL (log-transformed). Results reported as Too Numerous To Count (TNTC) are shown with grey stripes and set to the threshold value. In the O3 + GAC treatment, *Enterococci* counts were zero in both campaigns.

Six different bacterial indicators, relevant for water quality and reuse applications, were analysed using culture-based methods. The indicators include total aerobic bacteria at 22 °C and 37 °C, coliforms, faecal coliforms, *Escherichia coli*, and *Enterococci*. Analyses were performed during campaigns C2-I and C10-I on the effluents of the quaternary treatments (CW, GAC, and O3 + GAC) (Figure 11, Table S13). Except for *E. coli* and *Enterococci*, CW effluent shows lower concentrations of bacteria compared to the other treatments.

#### 3.3.5. Total and Viable Cell Counts

**Figure 12:**
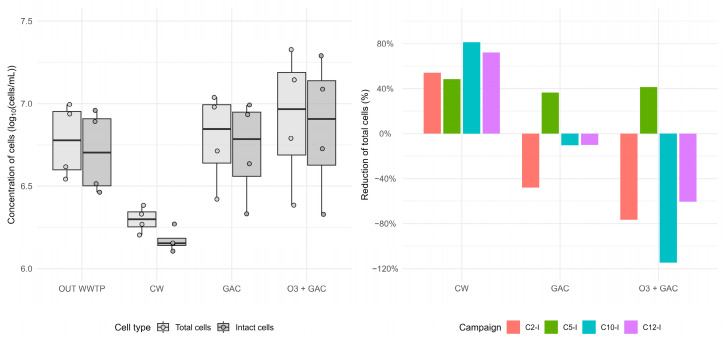
Cell concentration evaluation using flow cytometry expressed as (**Left panel**) total and intact cell counts in wastewater from the OUT WWTP and the three quaternary treatments (CW, GAC, and O3 + GAC) across the four intensive campaigns, expressed as cells/mL (log-transformed); (**Right panel**) percentage reduction in total cell counts (TCCs) in CW, GAC, and O3 + GAC relative to the OUT WWTP reference sample across the four campaigns.

Total cell counts (TCCs) were measured in OUT WWTP, CW, GAC, and O3 + GAC samples (Figure 12—left panel; Table S14). Total cell removal in CW was higher for all campaigns (63.9 ± 15.3% reduction) compared to GAC and O3 + GAC treatments (Figure 12—right panel). In GAC and O3 + GAC treatments, total cell concentrations even increased (+10 to +115%) for three of the four campaigns, indicating a release of cells during the treatment.

The number of viable cells was calculated as the total cell count (cells/mL) multiplied by the proportion of intact cells (%). The CW treatment consistently yielded lower numbers of intact cells across all four intensive campaigns (1.28 × 10^6^ to 1.86 × 10^6^ cells/mL). In contrast, both the GAC (2.15 × 10^6^ to 9.81 × 10^6^ cells/mL) and O3 + GAC (2.13 × 10^6^ to 1.95 × 10^7^ cells/mL) treatments exhibited the greatest variability between campaigns. In the OUT WWTP samples, viable cell counts ranged from 2.91 × 10^6^ to 9.09 × 10^6^ cells/mL.

#### 3.3.6. Metagenomics Campaign C12-I (CW and OUT WWTP)

Sixty-three MAGs were identified in the OUT WWTP sample, whereas forty-four MAGs were found in the CW sample (C12-I campaign). Completeness of the MAGs was relatively low, with eight non-contaminated MAGs with ≥50% completeness identified for the OUT WWTP sample and one MAG for the CW sample (Figure S3). The taxonomy of all the identified MAGs resulted in five different genera (for a total of seven MAGs) for CW and fifteen different genera (for a total of twenty-four MAGs) for OUT WWTP. Among others, *Flavobacterium* and *Rhodoferax* for the OUT WWTP sample and *Nitrospira* for the CW sample were found both in metabarcoding and metagenomic analyses. ARGs were still investigated on contigs from both samples. Six distinct ARG categories, corresponding to fourteen different genes, were detected in the OUT WWTP sample (*sul*, *qac*, *lnu*, *tet*, *mph*, *msr;* Table S15), while four categories, corresponding to five genes, were found in CW (*bla*, *sul*, *erm*, *lnu*; Table S16).

## 4. Discussion

The following discussion will address the advantages and disadvantages of constructed wetlands (CWs) as quaternary post-treatment, in comparison with granular activated carbon (GAC) and a combination of ozonation and activated carbon adsorption (O3 + GAC) applied to the urban wastewater treatment plant (rural environment; UWWTP 13.000 p.e.).

### 4.1. Compliance with Minimum Requirements for Water Reuse

Results from this study indicate that the effluents produced by CW and O3 + GAC were in compliance with the requirements for watering “food crops consumed raw, where the edible part is cultivated above ground and is not in direct contact with reclaimed water, processed food crops, and non-food crops, including crops for feed for milk- and meat-producing animals” (Reuse Category B; EU 2020/741), as *E. coli* concentrations remained below 100 CFU/100 mL. Interestingly, the effluent after GAC treatment was non-compliant, with concentrations of *E. coli* remaining above 50 (C5-I) and 5000 (C10-I) CFU/100 mL [65]. To confirm the categorisation of the reuse category, more precise results (<TNTC) would be needed. These unexpected results can be explained by the experimental setup (without pre-filtration) and the limitation of the automation (no automatic backwash) not being in line with conventional operation procedures. These results show the potential of CW to produce effluent with high quality and versatility. The implementation of CW for reuse applications (alone or combined with other treatments) is a topic that is currently under evaluation, yet its feasibility/applicability remains to be proven and to be recognised by the relevant authorities and water managers [66,67].

### 4.2. Heterogenous Fate of Antibiotics During Wastewater Treatment

Removal of the antibiotics CLA and SMX, as well as the SMX metabolite N-SMX, was investigated. When evaluating the performance of the WWTP for antibiotic removal (Figure 4), contrasting behaviours were observed. On one hand, CLA showed a negative (median) removal of −23.5%, meaning a poor removal in the biological process. As macrolides are known to adsorb in the activated sludge during the treatment, a low elimination can be attributed to the accidental release of sludge experienced during rainy-weather conditions in Bliesen. Additionally, the measurement of CLA was affected by high analytical uncertainties with different recovery rates/LOQs in influent (68%) and effluent (39%), which made the determination of the already small concentration even more vulnerable. On the other hand, SMX was removed by 66.3%, and its metabolite N-SMX exhibited an elimination of 93.4%. With N-SMX being a metabolite of SMX, when combined, a reduction of 89.6% was observed. These results are in line with studies that use high sludge retention times [68,69,70].

Contrasting behaviours in antibiotic removal were also seen for the quaternary treatments. CLA concentrations decreased consistently by 90% for any of the three quaternary treatments applied (38.1 to ~3.8 ng/L) and thus helped in the removal of CLA by the WWTP even though it already matched EQS values (0.13 µg/L) defined for receiving surface water. Removal of SMX by GAC and O3 + GAC treatments was also higher than 80%. In the CW, however, a release of SMX was observed (23.5 to 44 ng/L). This phenomenon was not observed in the case of N-SMX (~80%). Table S17 presents a comparative overview of the results obtained in this study and those reported in the literature regarding ATB and ARG removal rates in CWs, specifically in vertical-flow CWs (VF-CWs) and urban wastewater.

Our results highlight the heterogeneous fate of antibiotics within conventional activated sludge treatment and in CWs, with some compounds persisting in treatment, and others being more easily removed, due to compound-specific mechanisms such as sorption, biodegradation, metabolite transformation, and/or secondary release. In particular, the conversion of sulfonamides into acylated forms and their reversion to their parent compound during sludge waste treatment has previously been reported [50]. This reversible transformation may explain the abundance of SMX in the WWTP effluent, and the higher removal observed for N-SMX compared to the non-acetylated form.

### 4.3. Antimicrobial Resistance Propagation

The presence of antibiotics in treated wastewater may promote the development, propagation, and spread of antimicrobial resistance within the microbial communities present in the receiving water body [71]. Mobile genetic elements, such as the class 1 integrase gene cassette (*intI1*), are known to harbour ARGs and are thus likely to contribute to the spreading of ARGs among microbial communities via horizontal gene transfers. Therefore, several genes involved in resistance development and propagation of ARGs were monitored alongside the antibiotics to better evaluate treatment performance and assess the potential impact on antimicrobial resistance dissemination. To this aim, qPCR was selected as the key method to monitor ARGs in treated water because of their high specificity and sensitivity, independence regarding the physiological state of cells, and lower costs compared to culturing methods [10].

In general, in this study, the quaternary treatments reduced ARG (*blaAmpC*, *ermB*, *sul1*, and *tetW*) and *intI1* quantities more effectively than the WWTP alone (Figure 8), with lower concentrations in the treated water compared to that of the OUT WWTP (except for *intI1* in O3 + GAC). This is in line with the literature demonstrating the added value of quaternary treatments for the removal of ARGs and antimicrobial-resistant bacteria (ARB). Despite having the overall best removal (86%), O3 + GAC had difficulties removing *intI1,* suggesting a potential increase in gene transfer as found in another study. Overall ARG and *intI1* removal was next best in CW (68%) and last in GAC (15%).

The lower performance seen in GAC (when ARGs are released, Figure 8; when the total number of cells is higher, Figure 12) can be attributed to several factors. Firstly, the data are strongly influenced by the *tetW* results, with a release of the gene, rather than removal (−106%), which may be explained by the ability of activated carbon to accumulate cells and subsequently release them. Secondly, operational restrictions occurred, meaning that the GAC pilot was operated under constant loading with manual washing cycles (instead of time or pressure automation), and the potential saturation of the GAC was not assessed during the study (5381 bed volume reached). Despite the application of an EBCT of 61 min, these factors may underline the lower removal of ARGs and *intI1* seen in GAC and explain the differences with observations from the literature [11].

The metagenomic approach was used as an alternative to qPCR to describe a broader diversity of ARGs present in the treated water. Fourteen different ARGs were identified in total for both OUT WWTP and CW. In line with expectations, the four ARGs targeted with qPCR (*blaAmpC*, *ermB*, *sul1, tetW*) were also identified using metagenomics. An additional four genes not targeted by qPCR were identified; among them were genes involved in quaternary ammonium transport (*qac*), lincosamide resistance (*lnu*), and macrolide resistance (*mph, msr)*. This demonstrates the potential to detect the versality of ARGs without a priori knowledge of the genomes present in the community.

Given the low sequencing depth, CW’s efficiency in eliminating ARGs cannot be proven in this study. The only certainty is that both WWTP (*sul*, *qac*, *lnu*, *tet*, *mph*, *msr*; Table S15) and CW (*bla*, *sul*, *erm*, *lnu*; Table S16) are incapable of eradicating all AMR, since *lnu* and *sul* genes are detected in both effluents. The effectiveness of ARG removal by quaternary treatments requires deeper analyses.

For the four ARGs targeted with qPCR (*blaAmpC*, *ermB*, *sul1, tetW*), tetracycline resistance genes (*tet* genes) were not detected in the CW metagenomic bins, while four different *tet* genes were identified in the OUT WWTP sample. This observation is in line with data from qPCR, showing a 70.1% reduction from OUT WWTP to CW.

Interestingly, no genes corresponding to *intI1* were identified in OUT WWTP nor CW, which contrasts with the data observations from qPCR, especially regarding the OUT WWTP sample that showed a release of the *intI1* gene in the effluent. Technical limitations concerning the sequencing method may underline these observations (sequencing depth, quality of the DNA, etc.).

### 4.4. Considerations for Accurate Monitoring of ARG Removal

The literature presents contrasting perspectives on whether absolute ARG concentrations or relative abundances are more appropriate for evaluating ARG removal [71,72,73,74]. Most studies report only one of these metrics rather than both, making direct comparisons of plant performances challenging. Both metrics, however, provide valuable insights but may serve different purposes. Absolute concentrations are considered more relevant for evaluating individual plant performance and risk assessments, whereas relative abundances are considered more suitable for assessing removal across WWTPs (harmonisation of protocols), and/or to reveal selective processes within the plant such as potential horizontal gene transfer of the resistance genes [10,71]. Our study shows good performance for both metrics, and is in alignment with the recommended guidelines for ARG monitoring in surface water [10].

In addition to the necessity of the accurate presentation of the data, the importance of temporal scale for sampling cannot be overstated. Temporal fluctuations were observed among all quaternary treatments tested in this study and among multiple parameters. For example, an increase in ARGs and *intI1* concentrations was observed across all treatments from C2-I to C5-I, followed by a subsequent decrease from C5-I to C10-I. From C10- to C12-I, *blaAmpC* and *tetW* gene concentrations increased or stabilised, while *intI1* gene copy concentrations declined. Similarly, *ermB* and *sul1* gene copy concentrations decreased or stabilised in the GAC and O3 + GAC treatments, while the CW treatment tended to show an increase for those two genes between C10-I and C12-I. Removal of CLA was highly variable in time (from −218.5 to 40.0%) with very low inlet concentration (<200 ng/L for IN WWTP and <10 ng/L for OUT WWTP).

Temporal fluctuations may support the decision to work with annual means and/or the assessment of removal performance over longer periods of time with higher sampling frequencies, as opposed to assessing time-specific removal. However, it should be noted that averaging may mask some effects [71].

### 4.5. Microbial Perspective Through Bacterial Community Profiling

In parallel with the quantitative analysis of antibiotics, ARGs, and *intI1*, a more qualitative approach was applied to study the impact of the quaternary treatments on the microbial community by means of sequencing methods (16S rRNA gene barcoding). The ensuing discussion is founded upon an extensive review carried out by Wang et al. in 2022 [75], which provided a synopsis of the role microorganisms play in pollutant removal from wetlands [75]. Where relevant, additional references are cited.

It was observed in the present work that OUT WWTP and CW communities were not significantly different (*p* = 0.063), which aligns with previous studies, where it was reported that communities described in CWs are dominated by the same phyla as found in the WWTP effluent [75]. In contrast, communities from the GAC and O3 + GAC differed significantly from the OUT WWTP (*p* = 0.036 and *p* = 0.029, respectively). The O3 + GAC treatment induced a distinct and significant shift in the microbial community compared to GAC (*p* = 0.028), as described by Sun et al. [76].

Several bacterial genera have been reported to contribute to the removal of nutrients and antibiotics in constructed wetlands through biotransformation, biosorption, or related mechanisms [75]. In this study, genera associated with nitrification (*Nitrosomonas* and *Nitrospira*) were detected in OUT WWTP (0.15–1.26%, which are, respectively, minimum and maximum abundances across all campaigns) and CW (0–3.41%), while they were low or absent in GAC (<0.29%) and O3 + GAC (<LOD). These observations are in line with the extra ammonium removal measured in CW reaching almost complete elimination (99.8%; N-NH_4_ < 0.15 mg N/L), while this was not the case for GAC and O3 + GAC.

In contrast, nitrate concentrations increased in OUT WWTP and CW due to nitrification and incomplete denitrification. The partial denitrification process may occur and is in line with the presence of denitrifiers such as *Denitratisoma* (0.44–4.45% in CW), *Dechloromonas* (detected across all treatments) [75,77,78], and *Rhodoferax* (0–10.94% in CW). Physicochemical data confirm that incomplete denitrification occurred in CW because of insufficient biodegradable COD available for denitrification.

Extra phosphorus removal was measured in CW (97.6%; P_tot_ < 0.1 mg/L, except for C12-I) compared to GAC and O3 + GAC. Although in low abundance, this extra removal efficiency in CW coincided with the presence of phosphorus-accumulating organisms such as *Acinetobacter* (0–0.75% in CW), *Dechloromonas* (0–2.57% in CW), and members of *Rhizobiaceae* (0–0.22% in CW). The co-occurrence of these taxa in CW may provide favourable conditions for phosphorus elimination compared to the other treatments. The collective impact of phosphorus removal via (i) plant uptake, (ii) microbial transformation, and (iii) substrate accumulation are likely to contribute to the extra P removal that was observed [79].

Several studies report on CWs being highly suitable for antibiotic removal. Similarly to other pollutants, the removal of antibiotics involves a series of complex processes, such as adsorption, precipitation, and microbial degradation, the latter considered to be a key driver for their elimination. Indeed, several bacterial genera identified in this study have been described to play a role in antibiotic degradation, such as *Pseudomonas*, *Acinetobacter*, *Hyphomicrobium*, and *Acidovorax* [75]. *Pseudomonas* was present in all treatments (0.17–1.58% in CW; 0.49–19.83% in O3 + GAC; 0.68–4.65% in GAC), while *Acinetobacter* occurred in OUT WWTP (0.06–1.16%) and CW (0–0.75%) and was nearly absent in GAC and O3 + GAC. Additional taxa included *Hyphomicrobium* (0–0.59% in CW; up to 0.1% in O3 + GAC) and *Novosphingobium* (up to 0.11% in CW). The presence of these genera in CW and O3 + GAC may underlie the higher removal efficiencies of *tetW* (70–80% in CW; >95% in O3 + GAC) and *blaAmpC* (75–80% in CW; >95% in O3 + GAC). Conversely, GAC was dominated by *Acidovorax* (11.34–19.07%) and *Pseudomonas* (0.68–4.65%), both frequently reported as ARG carriers, which is in line with the lower ARG removal observed. For sulfonamide-related taxa, *Sphingobium* and *Hyphomicrobium* were found only in CW (0–0.32% and 0–0.59%, respectively) and at low levels in O3 + GAC (≤0.1%) [75,80]. The latter is however contradictory to the inadequate removal of SMX by CW, as presented previously. The contrasting observations may be partly explained by the fact that SMX biodegradation is influenced by environmental conditions (pH, temperature, nutrients, etc.) and by the presence of other microbial community members due to co-metabolism [81].

Although long-read metabarcoding provides merely semi-quantitative insights, the data reveal clear trends linking antibiotic degradation (sulfonamides) and ARG removal with microbial indicator taxa. Reduced antibiotic concentrations may lower the selective pressure for AMR development and thereby contribute to the reduction in ARGs in the effluents, while communities dominated by ARG carriers could impede this process. An in-depth correlation analysis (manuscript in preparation) is being carried out to demonstrate whether relationships between bacterial taxa (metabarcoding) and the removal of ARGs exist. Indeed, correlations were identified between microbial taxa and the presence of ARGs, concerning, for example, *ermB* (spread among phylogenetically diverse taxa), *tetW* and *sul1* (spread among phylogenetically restricted taxa), and environmental parameters (nitrification). Similarly, the gene copy concentrations of *sul1* and *intI1* were positively correlated, with coefficients of 78.2% (non-normalised data) and 79.7% (16S rRNA normalised data). This association, well-documented in the literature [82,83,84], is attributed to their genetic organisation, since *sul1* is located in the conserved region of class 1 integrase. These preliminary findings point towards potential co-occurrence and/or co-selection mechanisms, driven by treatment-specific characteristics regarding biodegradation pathways or adsorption dynamics in the treatment system.

## 5. Conclusions: Constructed Wetlands as a Resilient Alternative to Advanced Treatment

In the context studied, CWs proved to be a robust and reliable method to treat urban wastewater contaminated with micropollutants. In particular, the findings reinforce the added value of nature-based solutions as quaternary treatments for wastewater management in rural areas.

The results from this study highlight the following features of CWs:

- Generating effluent with excellent water quality regarding macropollutants, with particularly stable nitrogen and phosphorus removal. Water quality was compliant with the stringent standards imposed for medium and small WWTPs.
- Showing good performance regarding the removal of CLA by 90%, ensuring compliance with its EQS values and N-SMX (~80%). Negative removals were instead observed for SMX due to the possible reversible transformation of N-SMX in its parent compound.
- Demonstrating adequate and reliable removal of all ARGs and *intI1*, with low temporal variability compared to O3 + GAC and GAC. Only minor ARG release effects were observed in CW (e.g., *sul1* and *blaAmpC*). Although O3 + GAC achieved an average removal similar to that of CW, its inability to eliminate *intI1* underscored the comparative advantage of CW.
- Demonstrating the added value of CW for the removal of antibiotics. It underperformed regarding the removal of sulfamethoxazole, but not regarding its acetylated metabolite, showing the importance of monitoring transformation products.
- Producing effluent that is compliant with Reuse Category B standards—reuse water for applications including food crops consumed raw, processed crops, and non-food crops—as *E. coli* concentrations remained below 100 CFU/100 mL.

## Figures and Tables

**Table 1:** Primers used for qPCR analysis and corresponding thermal cycling conditions (modified from the literature).

Gene	Sequence (5′–3′)		Thermal Cycle		Reference
*blaAmpC*	Forward	CCTCTTGCTCCACATTTGCT	94 °C, 10 min	1× 40×	[46]
	Reverse	ACAACGTTTGCTGTGTGACG	94 °C, 30 s; 59 °C, 60 s; 72 °C, 30 s		
*ermB*	Forward	GATACCGTTTACGAAATTGG	94 °C, 10 min	1× 40×	[47]
	Reverse	GAATCGAGACTTGAGTGTGC	94 °C, 30 s; 59 °C, 60 s; 72 °C, 30 s		
*sul1*	Forward	CGCACCGGAAACATCGCTGCAC	94 °C, 10 min	1× 40×	[47]
	Reverse	TGAAGTTCCGCCGCAAGGCTCG	94 °C, 30 s; 59 °C, 60 s; 72 °C, 30 s		
*tetW*	Forward	GAGAGCCTGCTATATGCCAGC	94 °C, 10 min	1× 40×	[47]
	Reverse	GGGCGTATCCACAATGTTAAC	94 °C, 30 s; 59 °C, 60 s; 72 °C, 30 s		
16S rRNA	Forward	GGCTTCGTGATGCCTGCTT	95 °C, 10 min	1× 40×	[48]
	Reverse	GGWTACCTTGTTACGACTT	95 °C, 30 s; 56 °C, 30 s; 72 °C, 30 s		
*intI1*	Forward	GGGCGTATCCACAATGTTAAC	94 °C, 10 min	1× 40×	[46]
	Reverse	CATTCCTGGCCGTGGTTCT	94 °C, 30 s; 54 °C, 60 s; 72 °C, 30 s		

**Table 2:** Weather conditions, operational parameters, and physicochemical quality of WWTP effluent (temperature, °C; pH; conductivity, µS/cm; redox, mV).

Date	Campaign Duration	Season	Rainy or Dry Weather	Temperature	pH	Conductivity	Redox
20.1.22	24 h	Winter	Dry	10	6.8	542	364
24.2.22	72 h	Winter	Rainy	9	6.5	267	167
24.3.22	24 h	Spring	-	11	6.8	498	112
20.4.22	24 h	Spring	Dry	12	6.7	471	442
13.5.22	72 h	Spring	Rainy	16	6.7	526	492
25.5.22	24 h	Spring	Dry	16	6.4	269	522
16.6.22	24 h	Spring	Rainy	18	6.9	563	464
29.6.22	24 h	Summer	Dry	19	6.7	381	484
04.8.22	24 h	Summer	Dry	21	7.1	687	455
24.8.22	72 h	Summer	Dry	21	6.9	714	440
14.9.22	24 h	Summer	Dry	18	6.8	374	501
07.10.22	72 h	Autumn	Dry	15	6.8	435	485
17.10.22	24 h	Autumn	-	16	7.1	355	432

## Data Availability

The original contributions presented in this study are included in the article/Supplementary Materials. Further inquiries can be directed to the corresponding author(s).

## Supplementary Materials

The following supporting information can be downloaded at https://www.mdpi.com/article/10.3390/microorganisms13122663/s1. Protocol S1: Protocol used to create standard samples for qPCR analyses. Figure S1: (**Upper panel**) Gene copy concentrations of antibiotic resistance genes (*blaAmpC*, *ermB*, *sul1*, and *tetW*) and class 1 integrase gene (*intI1*) measured in the WWTP effluent (OUT WWTP), and after quaternary treatment (CW, GAC, O3 + GAC). Data are expressed as gene copies/L and log normalised. (**Lower panel**) Similar to data presented in the upper panel, except that the data is normalised by 16S rRNA gene concentrations; Figure S2: Alpha diversity indices for the OUT WWTP and the three quaternary treatments (CW, GAC, and O3 + GAC) across the four intensive campaigns (C2-I, C5-I, C10-I, and C12-I). (**Upper panel**) Species richness index; (**Middle panel**) Shannon index; (**Lower panel**) Simpson index showed as 1 − value so that the higher the value, the more diversity; Figure S3: Completeness (in %) of the MAGs from the 12th campaign; (**Upper panel**) for the CW sample; (**Lower panel**) for the OUT WWTP. MAGs’ taxonomy and contamination are represented by different colours as described in legends; Table S1: Requirements for discharges from urban wastewater treatment plant—macronutrient (10,000 to 150,000 p.e.); Table S2: Requirements for discharges from urban wastewater treatment plant—micropollutants (selection of a total of six in the First category (Fc) and two in the Second category (Sc)); Table S3: Weather conditions, operational parameters and physicochemical water quality; Table S4: Micropollutants measured—The micropollutant included in the UWWTD is highlighted with a star (*); Table S5: Wastewater volumes (in mL) filtered on 0.1 µm PES filter, used for DNA extraction. *^1^ Unanalysed sample (not received for analyses). *^2^; Filtration was carried out on 3 different filters to recover as many cells as possible. The volume mentioned is the total volume; Table S6: Elution volume (in µL) used to elute DNA after extraction (DNeasy® PowerWater® kit; Qiagen N.V. Germany). *^1^ Unanalysed sample (not received for analyses). *^2^; Filtration was independently done on the three filters with a 40 µL elution volume. All extracts were then pooled. The volume mentioned is the total volume; Table S7: DNA extraction concentrations (ng/µL) quantified with NanoDrop after DNA extraction (DNeasy® PowerWater® kit; Qiagen N.V. Germany). *^1^ Unanalysed sample (not received for analyses). *^2^; Filtration was independently done on the three filters. All extracts were then pooled. The concentration mentioned is the total concentration; Table S8: (Left) Mean measured values of physicochemical parameters measured across thirteen campaigns at different stages during the wastewater treatment. (Right) Calculated removal of the wastewater treatment plant alone and the removal quaternary treatment and WWTP combined in percentage; Table S9: Mean concentration and percentage removal of each micropollutants across all campaigns and standard deviation; Table S10: qPCR results expressed in gene copies per Liter of wastewater filtered (copies/L) for each target gene, for all lines and across the four intensives campaigns; Table S11: EMU abundance (16S rRNA barcoding) results for each treatment line (OUT WWTP and quaternary treatments) across four intensives campaigns; Table S12: EMU count (16S rRNA barcoding) results for each treatment line (OUT WWTP and quaternary treatments) across four intensives campaigns; Table S13: Heterotrophic plate counts (total aerobic bacteria at 22 °C and 37 °C, coliforms, faecal coliforms, *Escherichia coli*, and *Enterococci*) measured in the three quaternary treatments during the C5-I and C10-I campaigns; Table S14: Total cell count (TCC) and intact cell count (ICC) data acquired in flow cytometry for the WWTP effluent and the quaternary treatments across all intensive campaigns; Table S15: Antibiotic resistance genes detected in contigs from metagenomic analysis of the effluent sample at C12-I campaign; Table S16: Antibiotic resistance genes detected in contigs from metagenomic analysis of the CW sample at C12-I campaign; Table S17: Comparison of ATB and ARG removal performance in vertical-flow CW with values reported in the literature.

## Abbreviations

AMR	Antimicrobial Resistance
ATB	Antibiotic
ARB	Antimicrobial Resistant Bacteria
ARGs	Antibiotic Resistance Genes
AWaRe	Access, Watch, and Reserve Classification of Antibiotics
BOD	Biological Oxygen Demand
CLA	Clarithromycin
COD	Chemical Oxygen Demand
CW	Constructed Wetland
EBCT	Empty Bed Contact Time
EQS	Environmental Quality Standard
EU	European Union
GAC	Granular Activated Carbon
HLR	Hydraulic Loading Rate
ICC	Intact Cell Count
IQR	Inter-Quartile Range
LOD	Limit Of Detection
LOQ	Limit Of Quantification
MAG	Metagenome-Assembled Genome
MP	Micropollutant
N	Nitrogen
NH_4_	Ammonium
NO_2_	Nitrite
NO_3_	Nitrate
N-SMX	N-acetylsulfamethoxazole
O3 + GAC	Ozonation Followed by Granular Activated Carbon
ONT	Oxford Nanopore Technologies
P	Phosphorous
PCR (qPCR)	(Quantitative) Polymerase Chain Reaction
p.e.	Population Equivalent
PES	Polyethersulfone
SMX	Sulfamethoxazole
TCC	Total Cell Count
TSS	Total Suspended Solid
TNTC	Too Numerous To Count
TOC	Total Organic Carbon
UWWTD	Urban Wastewater Treatment Directive
UWWTP	Urban Wastewater Treatment Plant
VF-CW	Vertical-Flow Constructed Wetland
WWTP	Wastewater Treatment Plant
