# Supplementary material for: Fate and Removal of Antibiotics and Antibiotic Resistance Genes in a Rural Wastewater Treatment Plant: A Microbial Perspective of Nature-Based Versus Advanced Technologies
Source: Microorganisms. 2025 Nov 24;13(12):2663. doi: 10.3390/microorganisms13122663 (PMC12735332; doi:10.3390/microorganisms13122663)
Supplement: Supplementary file 1 [file microorganisms-13-02663-s001.zip › Supplementary-figures-CEBEDEAU.pdf]

microorganisms

MDPI

Protocol S1. Protocol used to create standard samples for qPCR analyses.

Standard samples for qPCR have been created specifically for each primer pairs. A DNA sample coming from a WWTP treating waters from a hospital and a veterinary clinic has been used. PCR using the primers has been conducted (using the same protocol as for the qPCR but with the GoTaq® Colorless Master Mix) followed by a migration of the PCR products on electrophoresis gels (E-Gel™ 2% agarose with SYBR™ Safe, Invitrogen, Thermo Fischer Scientific™, United States) to assess the selectivity of primer pairs for the targeted DNA fragment. PCR products have then been purified (MinElute® PCR purification Kit, Thermo Fischer Scientific™, United States), quantified (Quantus™ Fluorometer, Promega, United States) and finally diluted according to the equation:

$$\text{DNA copies}/\mu\text{L} = \frac{[\text{DNA}]_1 + [\text{DNA}]_2 + [\text{DNA}]_3}{3} \times \frac{\text{N}_\text{A}}{(\text{DNA}_{\text{lg}} \times \text{MM}_{1\ \text{bp}}) + \text{MM}_{\text{PO43}-}} \times 10^{-9}$$

where [DNA] is the DNA concentration in ng per µL, N_A is the Avogadro number, DNA_{lg} is the length of the targeted DNA fragment, $MM_{1\ bp}$ is the molecular mass of one base pair and MM_{PO43-} is the molecular mass of phosphate.

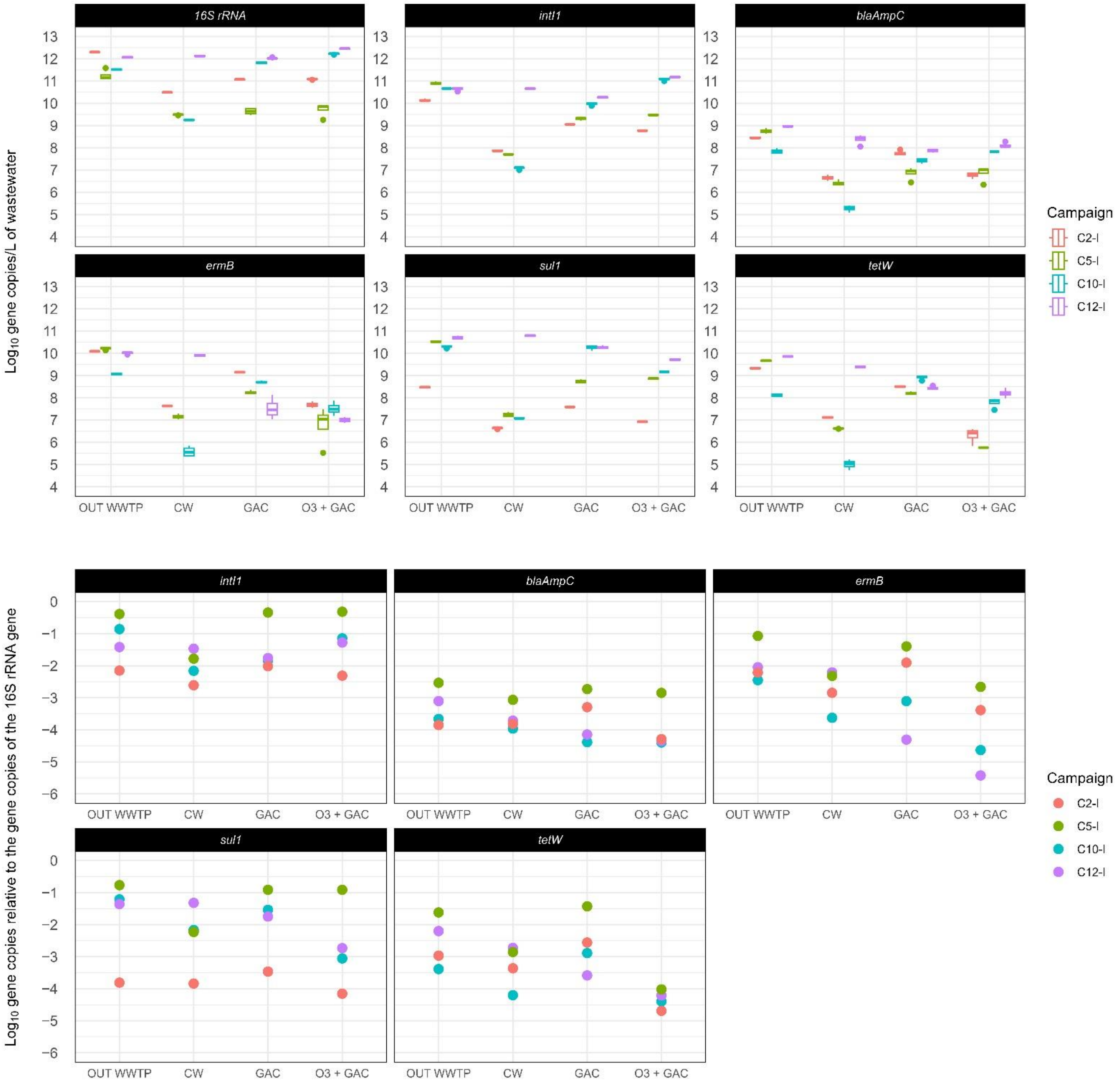

Figure S1. (**Upper panel**) Gene copy concentrations of antibiotic resistance genes (*blaAmpC*, *ermB*, *sul1*, and *tetW*) and class 1 integrase gene (*intI1*) measured in the WWTP effluent (OUT WWTP), and after quaternary treatment (CW, GAC, O3 + GAC). Data are expressed as gene copies/L and log normalised. (**Lower panel**) Similar to data presented in the upper panel, except that the data is normalised by 16S rRNA gene concentrations.

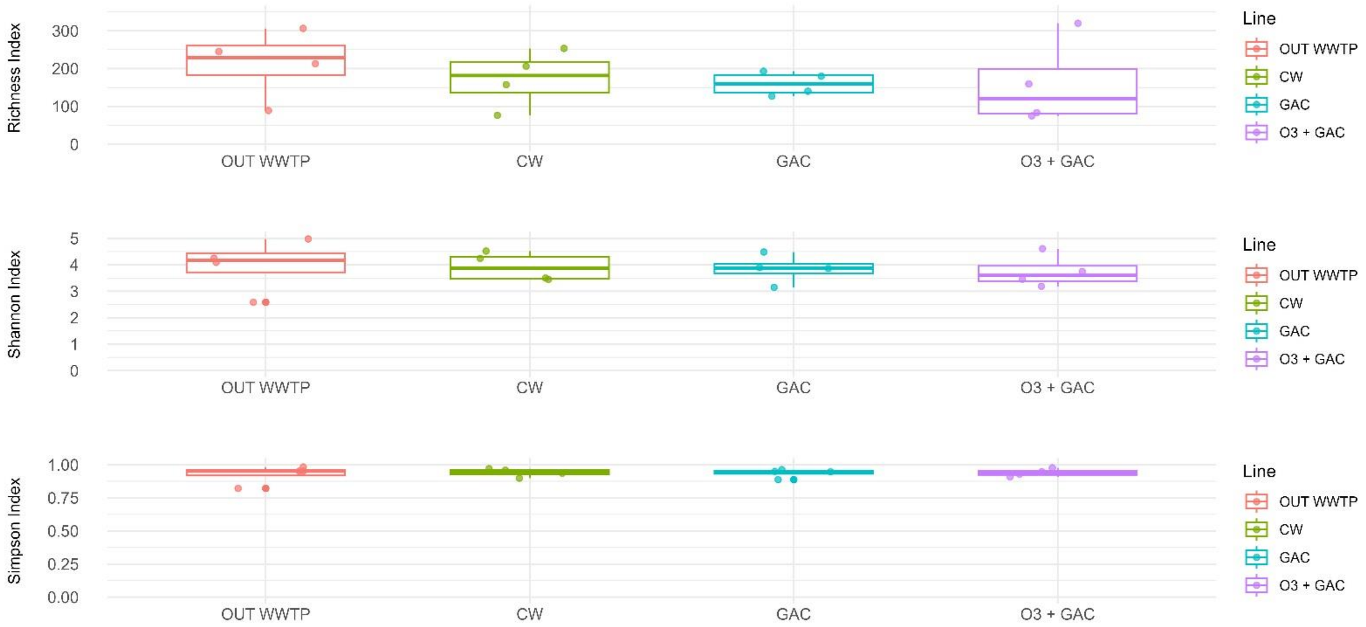

Figure S2. Alpha diversity indices for the OUT WWTP and the three quaternary treatments (CW, GAC, and O3 + GAC) across the four intensive campaigns (C2-I, C5-I, C10-I, and C12-I). (**Upper panel**) Species richness index; (**Middle panel**) Shannon index; (**Lower panel**) Simpson index showed as 1−value so that the higher the value, the more diversity.

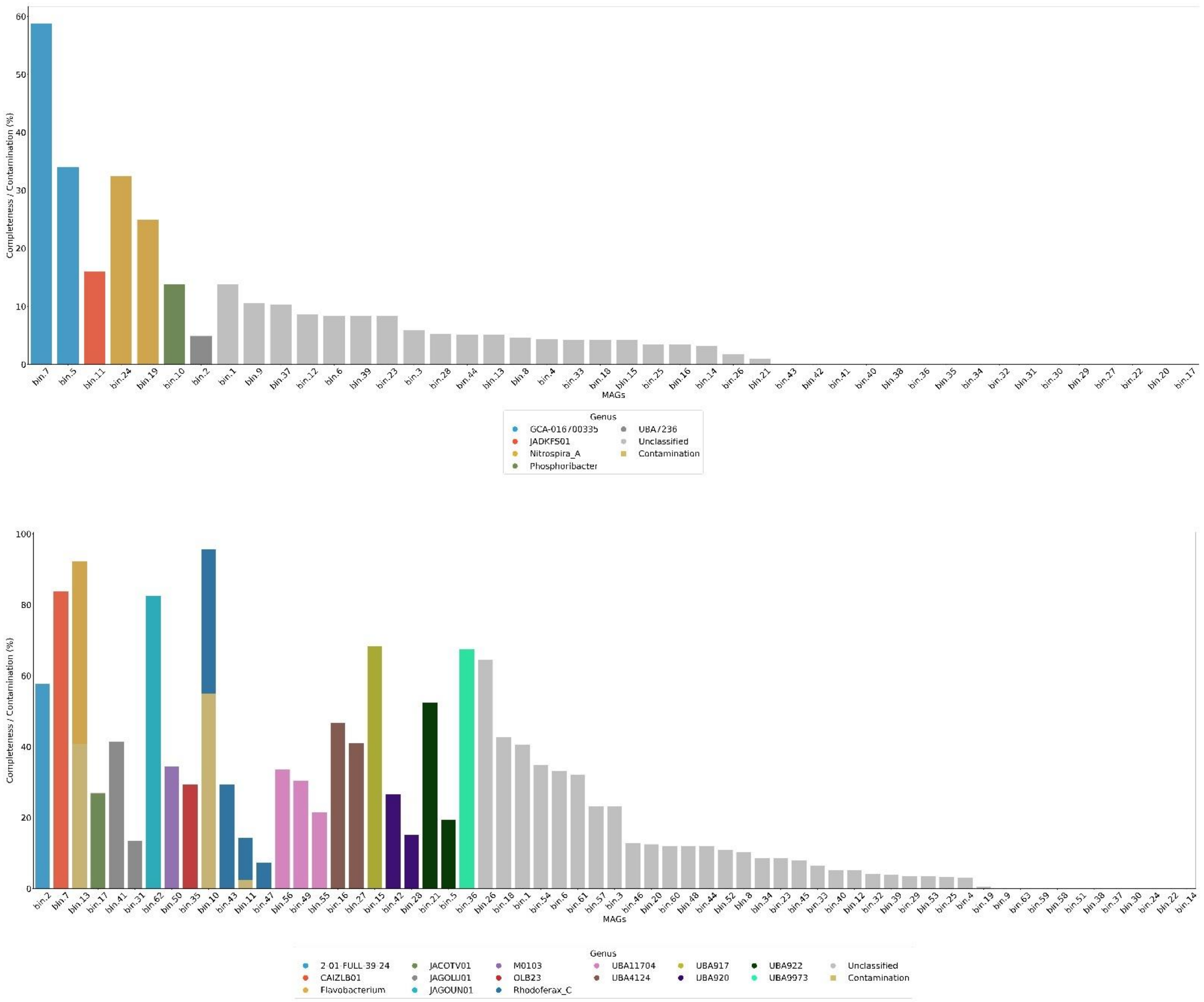

Figure S3. Completeness (in %) of the MAGs from the 12th campaign; (**Upper panel**) for the CW sample; (**Lower panel**) for the OUT WWTP. MAGs' taxonomy and contamination are represented by different colours as described in legends.
